# Heat hardening enhances metabolite-driven thermoprotection in the Mediterranean mussel *Mytilus galloprovincialis*

**DOI:** 10.3389/fphys.2023.1244314

**Published:** 2023-09-29

**Authors:** Ioannis Georgoulis, Christian Bock, Gisela Lannig, Hans O. Pörtner, Inna M. Sokolova, Konstantinos Feidantsis, Ioannis A. Giantsis, Basile Michaelidis

**Affiliations:** ^1^ Laboratory of Animal Physiology, Department of Zoology, School of Biology, Aristotle University of Thessaloniki, Thessaloniki, Greece; ^2^ Environmental Control and Research Laboratory, Region of Central Macedonia, Thessaloniki, Greece; ^3^ Alfred Wegener Institute, Helmholtz-Centre for Polar and Marine Research, Integrative Ecophysiology, Bremerhaven, Germany; ^4^ Department of Marine Biology, Institute of Biological Sciences, University of Rostock, Rostock, Germany; ^5^ Department of Fisheries and Aquaculture, University of Patras, Mesolonghi, Greece; ^6^ Department of Animal Science, Faculty of Agricultural Sciences, University of Western Macedonia, Kozani, Greece

**Keywords:** amino acids, 1 H-NMR spectroscopy, heat hardening, mussel, osmolytes, metabolomics

## Abstract

**Introduction:** Temperature affects organisms’ metabolism and ecological performance. Owing to climate change, sea warming constituting a severe source of environmental stress for marine organisms, since it increases at alarming rates. Rapid warming can exceed resilience of marine organisms leading to fitness loss and mortality. However, organisms can improve their thermal tolerance when briefly exposed to sublethal thermal stress (heat hardening), thus generating heat tolerant phenotypes.

**Methods:** We investigated the “stress memory” effect caused by heat hardening on *M. galloprovincialis* metabolite profile of in order to identify the underlying biochemical mechanisms, which enhance mussels’ thermal tolerance.

**Results:** The heat hardening led to accumulation of amino acids (e.g., leucine, isoleucine and valine), including osmolytes and cytoprotective agents with antioxidant and anti-inflammatory properties that can contribute to thermal protection of the mussels. Moreover, proteolysis was inhibited and protein turnover regulated by the heat hardening. Heat stress alters the metabolic profile of heat stressed mussels, benefiting the heat-hardened individuals in increasing their heat tolerance compared to the non-heat-hardened ones.

**Discussion:** These findings provide new insights in the metabolic mechanisms that may reinforce mussels’ tolerance against thermal stress providing both natural protection and potential manipulative tools (e.g., in aquaculture) against the devastating climate change effects on marine organisms.

## 1 Introduction

Seawater temperature increase due to climate change is a source of severe environmental stress affecting marine organism’s physiology and fitness (Pörtner, 2012; 2021). Extreme and rapid warming can exceed the physiological resilience and adaptive capacity of marine organisms. The latter may undermine their potential to respond and adapt to environmental changes (Doney et al., 2012; Pörtner, 2012; 2021). During exposure to moderate thermal stress, the metabolic scope is reduced due to the increased cost for basal maintenance, while extreme thermal stress causes limitation of uptake and delivery of oxygen to tissues, insufficient aerobic production of ATP and onset of the anaerobic metabolism to compensate for the increased energy demands (Pörtner, 2012; Sokolova, 2013; Eymann et al., 2020; Pörtner, 2021). Furthermore, exposure of ectotherms to supraoptimal temperatures leads to the disturbances in energy turnover, decreased mitochondrial coupling and excess reactive oxygen species (ROS) generation (Sokolova, 2023). Elevated temperatures also increase protein denaturation necessitating energy investment into the production and activity of molecular chaperones as well as degradation and replacement of damaged cellular proteins (Hawkins, 1995). These cellular phenomena lead to increased oxidative and proteotoxic stress and result in cellular and systemic damages that eventually increase organisms’ morbidity and mortality (Sokolova, 2013; 2018).

Several studies revealed that repeated exposure of an organism to sublethal thermal stimuli might modulate its response to future environmental conditions. This can be achieved by the acquisition of this experience’s memory, since it can result to phenotypic and stimulus-dependent plasticity of response traits (Hilker et al., 2016), which leads to the adjustment of physiological or developmental phenotype to thermal stress (Agrawal, 2001; West-Eberhard, 2003). The latter allows faster and more efficient response of the organism to subsequent stress re-exposure (Ding et al., 2012). “Heat-hardening” is used to describe this beneficial and rapid response that improves the organism’s heat tolerance when briefly exposed to sublethal temperatures and produces phenotypes which are more thermal stress-resistant (Precht, 1973; Bowler, 2005; Bilyk, et al., 2012). The pioneering work by Hutchison (1961) on the eastern newt *Notopthalmus viridescens* was among the first to demonstrate the organisms’ ability to increase their maximum critical temperature after exposure to sublethal heat stress. Similar heat hardening effects on heat resistance has been reported in various other species including the Mediterranean seagrass *Posidonia oceanica* (Pazzaglia et al., 2022)*,* frogs (Maness and Hutchison, 1980), fish (Menke and Claussen, 1982; Bilyk and DeVries, 2011; Bilyk, et al., 2012), insects (Huang et al., 2007; Marais et al., 2009), and bivalves such as *Perna canaliculus* (Dunphy et al., 2018), *Perna viridis* (Aleng et al., 2015) and *Mytillus galloprovincialis* (Georgoulis et al., 2021; Georgoulis et al., 2022).

The cellular mechanisms behind the enhanced organisms’ thermotolerance after heat hardening are not well known. Several studies reported that under stressful conditions (e.g., heat, cold or salt stress), organisms accumulate low-molecular-mass compounds, called organic osmolytes that help offset the potentially damaging stress effects (Rishi et al., 1998; Rabbani and Choi, 2018; Somero, 2022). Organic osmolytes (including methylamines, polyols and amino acids) can aid in thermal protection and stabilization of proteins through interactions with the protein surfaces that lead to regeneration of native protein forms from unfolded states (Hoffman et al., 2009; Rabbani and Choi, 2018). Furthermore, taurine, one of the most important organic osmolytes, contributes to the amelioration of thermal stress by protecting mitochondria, mitigating oxidative stress and ROS production and thereby contributing to macromolecular stability (Yancey, 2005; Sokolov and Sokolova, 2019). Studies on *M. galloprovincialis* have shown higher taurine levels associated with higher antioxidant defense in heat-hardened mussels, when comparing to the non-heat-hardened ones under elevated temperatures (Georgoulis et al., 2021; Georgoulis et al., 2022). The osmolyte pool’s modulation is relevant in both short-term and long-term acclimatization and therefore adaptive changes of osmolyte systems exhibit critical importance for rapid survival in warming (Somero, 2022).

Recent studies revealed that heat-hardened *M. galloprovincialis* displays improved cellular protection resulted from the metabolic reorganization and enhanced antioxidant capacity (Georgoulis et al., 2021; 2022). Specifically, heat-hardened mussels showed higher tricarboxylic acid (TCA) cycle activity, and at the same time diversification of upregulated metabolic pathways. The latter can be hypothesized to act as a mechanism increasing ATP production, in order to extend survival under heat stress (Sokolova, 2013). Moreover, taurine was accumulated, which may provide an antioxidant and cytoprotective role in mussels during hypoxia and thermal stress (Georgoulis et al., 2022), indicating that osmolytes might also be involved in cellular protection induced by the heat hardening. To examine the potential role of osmolytes in thermoprotection of the mussels, we examined the adaptive adjustments of the osmolytes in the heat hardened *M. galloprovincialis* compared to the non-heat-hardened individuals. To assess the relative shifts in osmolytes compared to other intermediary metabolic compounds, non-targeted metabolomic analyses was conducted using ^1^H-NMR spectroscopy.

## 2 Materials and methods

The animals used in the present study originate from the same exposures as described in earlier published studies (Georgoulis et al., 2021; Georgoulis et al., 2022). A brief description is provided in the following sections.

### 2.1 Animals

As described in Georgoulis et al. (2021), Georgoulis et al. (2022), *M. galloprovincialis* mussels with total mass 25.82 ± 4.62 g, shell length 6.42 ± 0.47 cm and shell width 3.2 ± 0.15 cm (mean ± SD) were collected in the end April 2021 from a mussel aquaculture farm which is located in Thermaikos Gulf, Greece. At the time of collection, the ambient sea water temperature was approximately 18°C. Thereafter, collected mussels were transferred to the Laboratory of Animal Physiology, Department of Zoology, School of Biology of the Aristotle University of Thessaloniki. There, they were transferred and kept in tanks with recirculating aerated natural sea water for 1 week. Temperature was kept at 18°C ± 0.5, salinity at 34‰ ± 2.85 and pH at 8.12 ± 0.05. Mussels were fed daily with cultured microalgae *Tisochrysislutea* (CCAP 927/14) at 0.5% dry mass of algae relative to the total mass of mussels.

### 2.2 Experimental procedures

Mussels were sequentially exposed to heat-hardening and acclimation phases as previously described in Georgoulis et al. (2021).

i Heat-hardening phase

The group of mussels which is later referred as a heat-hardened (H), was exposed to the heat-hardening conditions. The heat-hardening experimental design was based on Hutchison’s “Repeated—critical thermal maximum (CTM)” method (Hutchison, 1961). In brief, ∼300 randomly selected mussels were conditioned in three replicate aquaria. Each aquarium contained 100 L aerated sea water and the temperature was kept at 18°C for 1 week. Thereafter, water temperature was increased to 27°C and maintained at this level for 2.5 h. The rate of temperature increase was set to 1.5^°^C/min. Then, water temperature decreased again to the control temperature of 18°C (1.5^°^C/min). Mussels were left to recover at this temperature for 24 h. This heat-stress bout, both the thermal shock phase and the recovery phase, was repeated four times.

ii Acclimation phase

After the heat-hardening treatment, both non-hardened (group NH) and heat-hardened mussels (group H) were transferred to four 500 L tanks (50–60 individuals from each group per tank) with recirculating aerated natural sea water at 18°C and left to recover for 4 days. Within each tank, both groups (NH and H) were placed in separate baskets. Thereafter, water temperature of the three tanks was increased by a rate of 1°C/h to 24°C, 26°C, and 28°C, respectively. The choice of the aforementioned was based on the following: warming of *M. galloprovincialis* beyond 24°C resulted to increased oxidative stress, energy misbalance, metabolic depression and a shift to anaerobic metabolism (Feidantsis et al., 2020). Moreover, acclimation beyond 26°C activates the heat shock response and the antioxidant defence in the same species, while at the same time it leads to increased mortalities (Anestis et al., 2007; Feidantsis et al., 2020; 2021; Georgoulis et al., 2021). In general, previous studies have shown that *M. galloprovincialis* is thermally stressed when exposed to 26°C–27°C (Anestis et al., 2007; Feidantsis et al., 2020). Although the most extreme heat stress for intertidal species usually occurs during emersion, it should be pointed out that ambient sea surface water temperature recorded in Northern Greece during July–August varies between 27 and 28.5°C (Feidantsis et al., 2018).

Mussels maintained at 18°C were used as controls to evaluate possible temporal effects on the parameters of interest. All exposure conditions were run in triplicates.

### 2.3 Tissue sampling and water quality monitoring

Individuals (n = 8 at each time point) from H and NH groups were collected from each tank after the desired temperature (24°C, 26°C or 28°C) has been reached, at the following time points: 12 h and 1, 5, 10 and 15 days At 24°C, both H and NH mussels showed no mortality, while at 26°C the mortality of NH mussels reached 50% and that of H ones 40%. However, due to the 100% mortality of NH mussels by the 10th at 28°C, samplings on the 15th day were not performed. We have to underline that contrary to the above, the H mussels exhibited a 45% mortality by the 15th day (Georgoulis et al., 2021; 2022). After mussels dissection, mantle tissue was removed and immediately frozen in liquid nitrogen. Then, it was stored at −80°C for later analyses. Mantle was chosen for this study since it exhibits higher aerobic capacity and more intense physiological stress response compared to other tissues. Additionally, several previous studies in mussels examined this tissue and thus it provided us feasible comparisons (e.g., Feidantsis et al., 2020 and references therein). Physicochemical water parameters were measured daily including salinity (g L^−1^), O_2_ (mg L^−1^) and pH by using Consort C535, Multiparameter Analysis Systems (Consort, bvba, Turnhout, Belgium), and concentrations of NH_3_ (μg L^−1^), NO_2_^−^ (μg L^−1^) and NO_3_^−^ (μg L^−1^) using commercial kits (Tetra Werke, Melle, Germany) (Table 1) (Georgoulis et al., 2021).

**TABLE 1:** Mean values of seawater parameters.

(°C)	TEMPERATURE (°C)	Salinity (G^−1^)	PH	O_2_ (MG L^-1^)	NH_3_ (MG L^−1^)	NO^2−^ (MG L^−1^)	NO^3 –^ (ΜG L^−1^)
18	18 ± 0.1	33.2 ± 0.03	8.04 ± 0.02	7.9 ± 0.06	0.2 ± 0.01	0.2 ± 0.01	<12.5
24	24 ± 0.3	33.4 ± 0.03	8.03 ± 0.02	7.8 ± 0.07	0.2 ± 0.01	0.3 ± 0.02	14 ± 0.6
26	26 ± 0.4	33.3 ± 0.01	8.02 ± 0.01	7.8 ± 0.05	0.2 ± 0.02	0.3 ± 0.01	14 ± 0.3
28	28 ± 0.3	33.2 ± 0.04	8.02 ± 0.01	7.8 ± 0.07	0.3 ± 0.02	0.2 ± 0.02	13 ± 0.5

### 2.4 Tissue extraction

A methanol chloroform extraction protocol was used for the mantle tissue (Rebelein et al., 2018; Georgoulis et al., 2022) Briefly, homogenization of about 100 mg of the frozen mantle tissue was performed in a solution containing 400 μL ice-cold methanol and 125 μL ice-cold Milli-Q water. The samples were centrifuged (20 s, 6,000 rpm) and thereafter the addition of 400 μL ice cold chloroform and 400 μL ice-cold Milli-Q water followed. After mixing and incubation on ice (10 min), samples were centrifuged (10 min, 3,000 *g*, 4°C) resulting in three phases: the upper methanol layer with the polar metabolites (which was transferred to a new tube and dried in a vacuum centrifuge at room temperature overnight), the lower chloroform layer with the lipids (which was dried in a fume hood at room temperature) and a thin layer of proteins in the middle. The samples’ water-soluble fractions which contained the cytosolic metabolites were transferred to the AWI in Bremerhaven. There, for metabolomic studies, they were dried at room temperature using a rotational vacuum concentrator (RVC 2–18 HCl, Christ GmbH, Germany).

### 2.5 Metabolomic profiling based on^1^H-NMR spectroscopy

For the untargeted metabolite profiling, 8 preparations from heat-hardened (H) and non-heat-hardened (NH) groups of animals at each time point were employed. This profiling was based on NMR spectroscopy, and was performed according to previous studies (Götze et al., 2020; Georgoulis et al., 2022). The NMR spectroscopy measurements were carried out on a vertical 9.4T wide bore magnet operating at 400.13 MHz with Avance III HD electronics (Bruker GmbH, Germany). Deuterated water (D_2_O) containing 0.05% thrymethylsilyl propionate (TSP, Sigma-Aldrich, USA) was employed in order to dissolve the dried tissue extracts (internal standard with a 1:1 ratio) which were thereafter transferred into 1.7 mm NMR tubes. Samples were then placed in a 1.7 mm diameter triple tuned (^1^H-^13^C-^15^N) probe. Thereafter, measurement with the employment of a classical Carl-Purcell-Meiboom-Gill (Bruker protocol cpmgpr1d) sequence with water suppression at room temperature followed. Measurement parameters were as follows: acquisition time (AQ) 4.01 s, sweep width (SW) 8802 Hz (22 ppm), delay (D1) 4 s, dummy scan (DS) 4, number of scans (ns) 256.

Chenomx NMR suite 8.1 (ChenomxInc, Canada) was used to determine metabolite profiles and concentrations. Therefore, the ^1^H-NMR spectra were phase and baseline corrected and set to the chemical shift of the TSP standard (0.0 ppm). The TSP signal served as line width and concentration standard (2.3 mM). Metabolites were identified using their corresponding NMR signals from the Chenomx database and profiled to the NMR spectrum of each individual spectrum for quantification.

### 2.6 Statistics

Significant changes in metabolites’ levels between examined groups were tested using one-way analysis of variance (ANOVA) (GraphPadInstat 3.0) at the 5% level (*p* < 0.05). Two-way (GraphPad Prism 5.0) ANOVA was employed for testing the effects of exposure time and treatment (H and NH mussel groups) as fixed factors. Post-hoc comparisons were performed using the Bonferroni test. Values are presented as means ± S.D.

MetaboAnalyst 5.0 was employed to analyze the differences in metabolite profiles and concentrations (Chong et al., 2019). Metabolite concentrations were transformed and scaled in order to achieve normalization. ANOVA and multi-variate statistics including principal component analysis (PCA) implemented in MetaboAnalyst were used to test for differences in normalized concentrations. Heatmaps and dendograms were performed within Metaboanalyst for an overview of metabolite patterns and hierarchical clusters.

## 3 Results

### 3.1 Branched-chain amino acids

**FIGURE 1:**
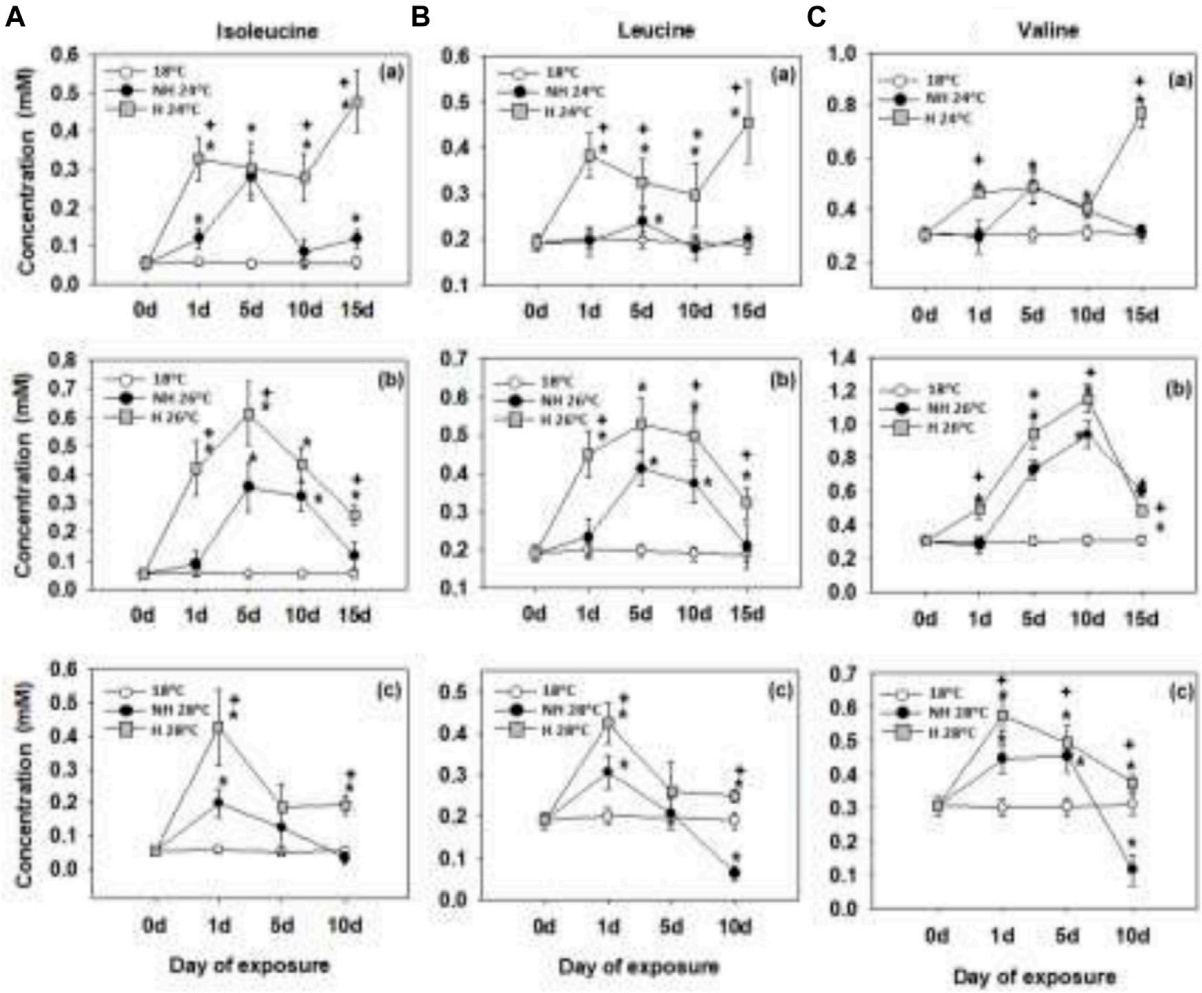
Isoleucine **(A)**, leucine **(B)** and valine levels in the mantle of heat-hardened (H) and non-heat-hardened (NH) *Mytilus galloprovincialis* mussels after exposure to 24°C **(a)**, 26°C **(b)** and 28°C **(c)** in comparison to acclimated at 18°C individuals (control). Values depict means ± SD of n = 8. Asterisk (*) depicts statistically significant changes (*p* < 0.05) when compared to control mussels, while cross (^+^) depicts statistically significant changes (*p* < 0.05) when compared to non-heat-hardened (NH) mussels.

The three examined BCAAs (valine, leucine and isoleucine) showed a sharp increase in their levels in the mantle tissues during 1–5 days of warming. The BCAA accumulation occurred earlier and reached higher levels in the H mussels compared to the NH ones. The elevated BCAA levels tended to persist longer in the H mussels compared to the NH ones, particularly at 24°C and 26°C (Figure 1).

### 3.2 Aromatic amino acids

**FIGURE 2:**
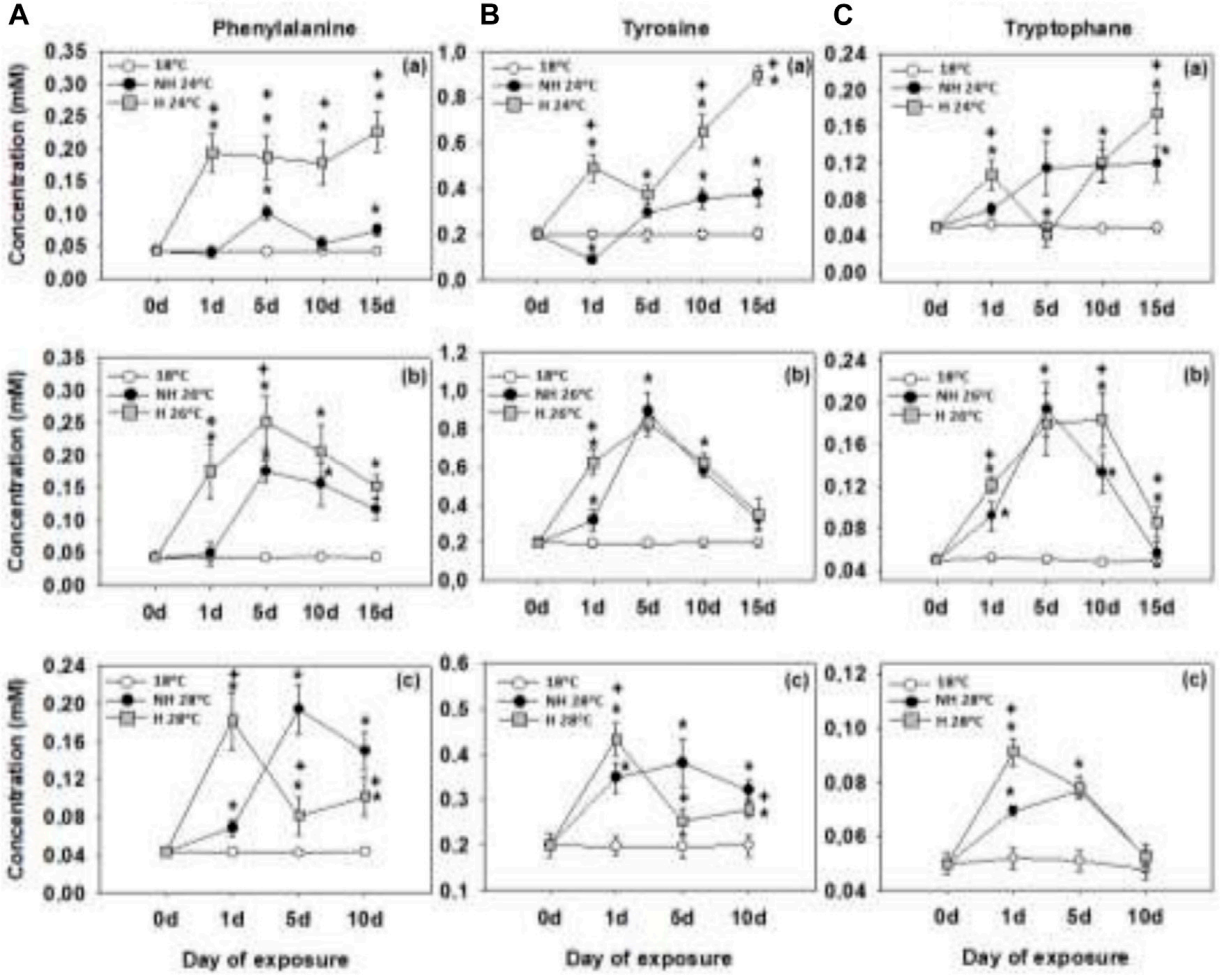
Phenylalanine **(A)**, tyrosine **(B)** and tryptophane **(C)** levels in the mantle of heat-hardened (H) and non-heat-hardened (NH) *Mytilus galloprovincialis* mussels after exposure to 24°C **(a)**, 26°C **(b)** and 28°C **(c)** in comparison to acclimated at 18°C individuals (control). Values depict means ± SD of n = 8. Asterisk (*) depicts statistically significant changes (*p* < 0.05) when compared to control mussels, while cross (^+^) depicts statistically significant changes (*p* < 0.05) when compared to non-heat-hardened (NH) mussels.

Warming at 24°C led to an increase in the tissue levels of phenylalanine, tyrosine and tryptophan in both NH and H mussels. The increase of phenylalanine and tyrosine concentrations was more pronounced in the H mussels, whereas in the case of tryptophan levels, no consistent differences were found between the experimental treatment groups (Figure 2Aa, Ba, Ca). At 26°C and 28°C, AAA levels transiently peaked after 1–5 days of exposure and then gradually decreased to the baseline (control) levels. We found no consistent differences in AAA concentrations between the H and NH mussels at 26°C and 28°C (Figure 2Ab; 2Ac; 2Bb; 2Bc; 2Cb; 2Cc).

### 3.3 Glutamine family amino acid metabolism

**FIGURE 3:**
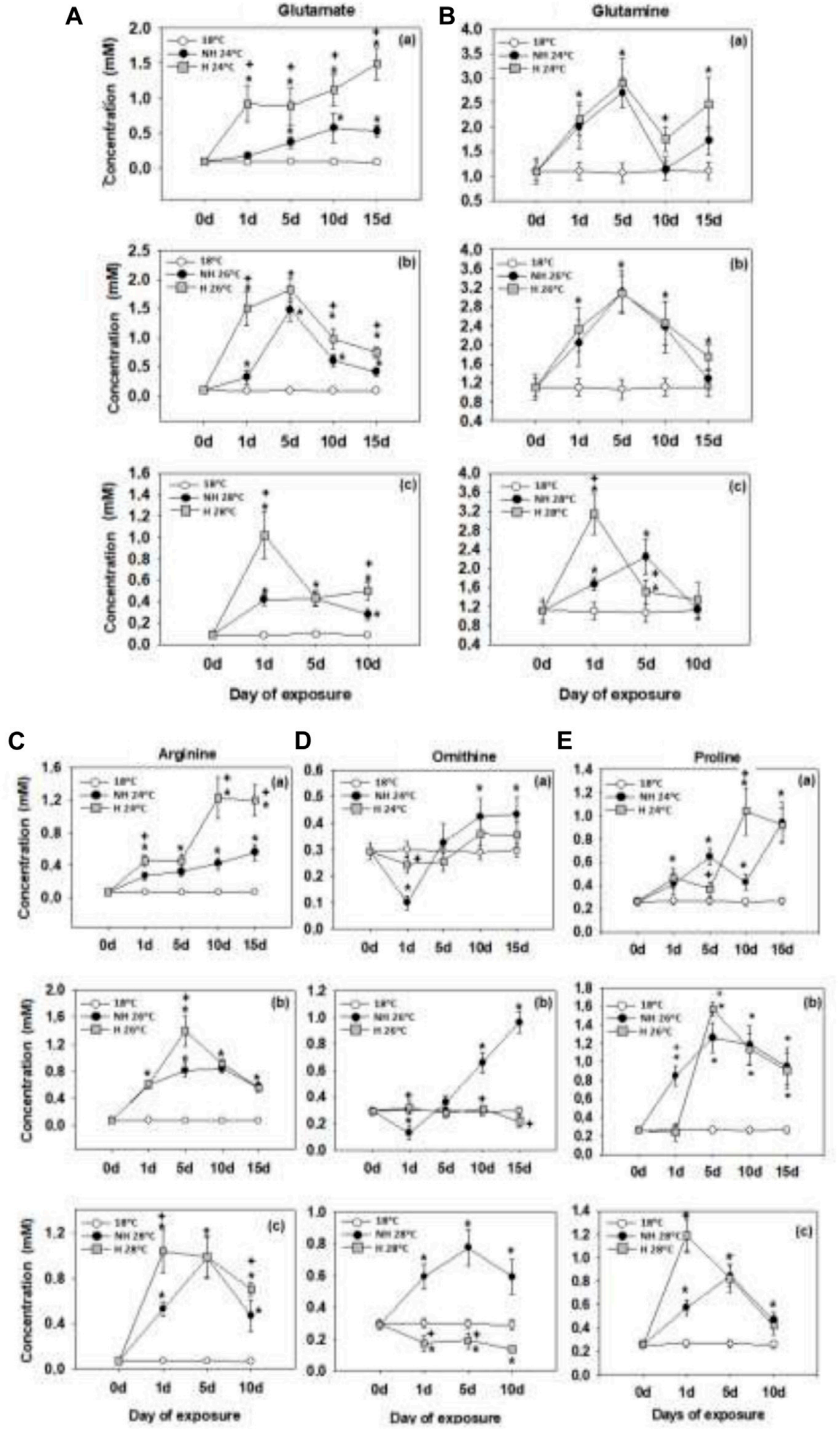
Glutamate **(A)**, glutamine **(B)**, arginine **(C)**, ornithine **(D)** and proline **(E)** levels in the mantle of heat-hardened (H) and non-heat-hardened (NH) *Mytilus galloprovincialis* mussels after exposure to 24°C **(a)**, 26°C **(b)** and 28°C **(c)** in comparison to acclimated at 18°C individuals (control). Values depict means ± SD of n = 8. Asterisk (*) depicts statistically significant changes (*p* < 0.05) when compared to control mussels, while cross (^+^) depicts statistically significant changes (*p* < 0.05) when compared to non-heat-hardened (NH) mussels.

Glutamate and glutamine levels exhibited a significant increase within the first 5 days of warming (Figures 3A,Β). The H mussels showed greater accumulation of glutamate compared to the NH ones, whereas increase in the glutamine levels was similar in both experimental groups (Figure 3A). The only exception to the latter pattern was a peak in glutamine concentration in the H mussels after 1 day of exposure to 28°C that greatly exceeded levels found in the NH counterparts (Figure 3Β).

Furthermore, experimental warming led to an increase in the concentrations of arginine, ornithine and proline in the mantle of mussels (Figures 3C–E). Generally, the concentrations of these amino acids reached peak values earlier with increasing temperatures [by 10–15 days at 24°C, 5 days at 26°C (except ornithine) and 1–5 days at 28°C]. Accumulation of ornithine was higher in the NH mussels than in the H groups, particularly at warmer temperatures and prolonged exposures (Figure 3D). In contrast, arginine concentrations reached higher levels in the H mussels than in their NH counterparts (Figure 3C). Proline concentrations in the mantle peaked earlier and at a higher level in the H mussels compared to the NH ones but were similar in the two experimental groups at other time points (Figure 3D).

### 3.4 Choline and related metabolites

**FIGURE 4:**
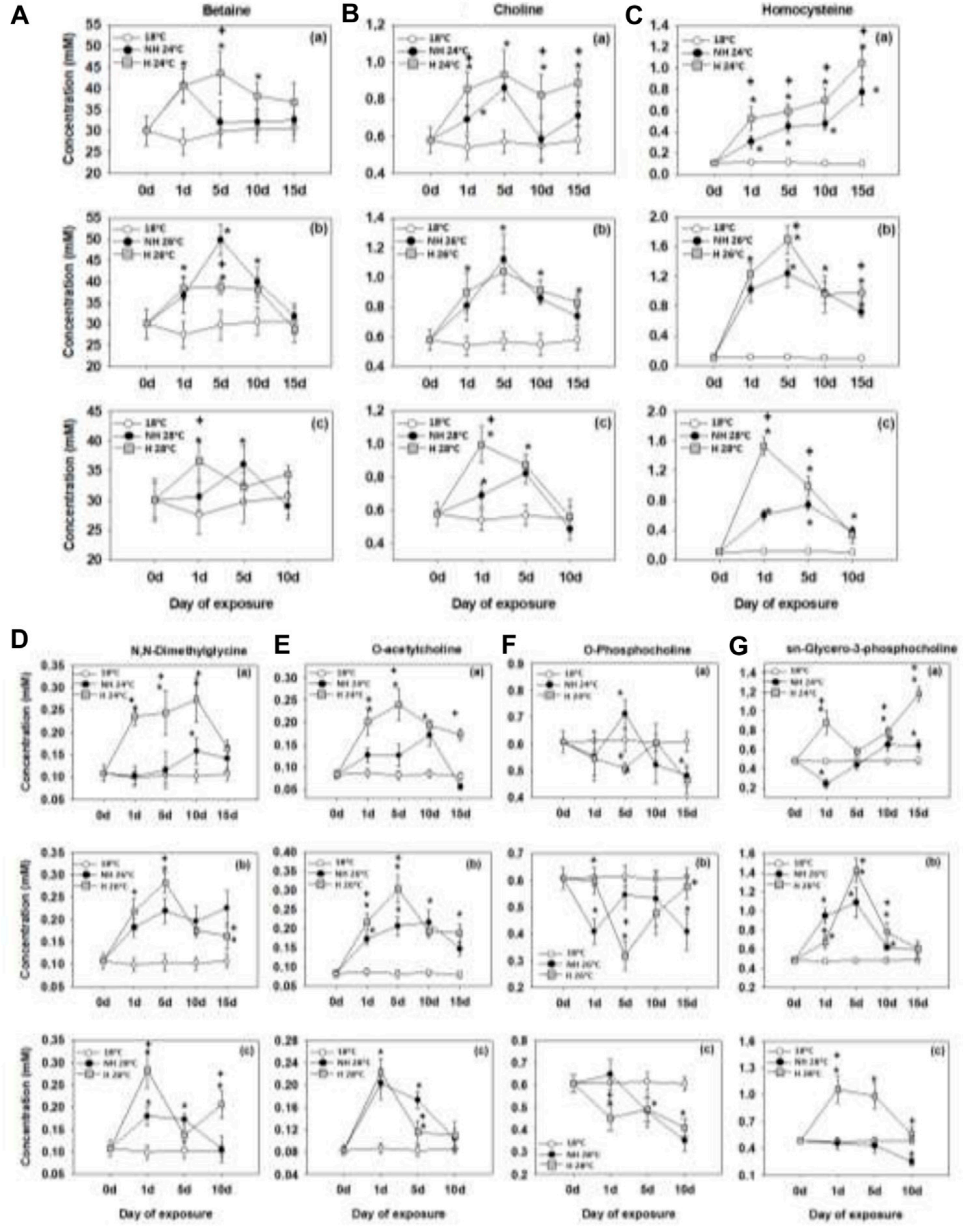
Betaine **(A)**, choline **(B)**, homocysteine **(C)**, N,N-dimethylglycine **(D)**, O-acetylcholine **(E)**, O-phosphocholine **(F)** and sn-glycero-3-phosphocholine **(G)** levels in the mantle of heat-hardened (H) and non-heat-hardened (NH) *Mytilus galloprovincialis* mussels after exposure to 24°C **(a)**, 26°C **(b)** and 28°C **(c)** in comparison to acclimated at 18°C individuals (control). Values depict means ± SD of n = 8. Asterisk (*) depicts statistically significant changes (*p* < 0.05) when compared to control mussels, while cross (^+^) depicts statistically significant changes (*p* < 0.05) when compared to non-heat-hardened (NH) mussels.

Warming induced elevated levels of betaine, choline and homocysteine in the mussels’ mantle tissue peaking after 5 days of warming (except homocysteine at 24°C reaching the highest levels after 15 days). Levels of these metabolites were higher in the H mussels compared with the NH ones at 24°C and 28°C (Figure 4). At 26°C, the pattern of differences in betaine, choline and homocysteine concentrations between the H and NH mussels was less consistent (Figure 4).

Mantle levels of N,N-dimethylglycine, O-acetylcholine and sn-glycero-3-phosphocholine showed a significant increase during warming. With a few exceptions, the levels of N-dimethylglycine, O-acetylcholine and sn-glycero-3-phosphocholine were higher in the H mussels compared with their NH counterparts. The mantle levels of these three metabolites reached the peak values earlier at the high temperatures (26°C and 28°C) than at 24°C (Figure 4D; 4E; 4G).

Concentrations of O-phosphocholine decreased during warming, particularly at the two higher exposure temperatures (26°C and 28°C) (Figures 4Fb, Fc). No consistent differences in the warming-induced shifts in O-phosphocholine concentrations were found between the H and NH mussels (Figure 4F).

### 3.5 Organic osmolytes and cytoprotective compounds

**FIGURE 5:**
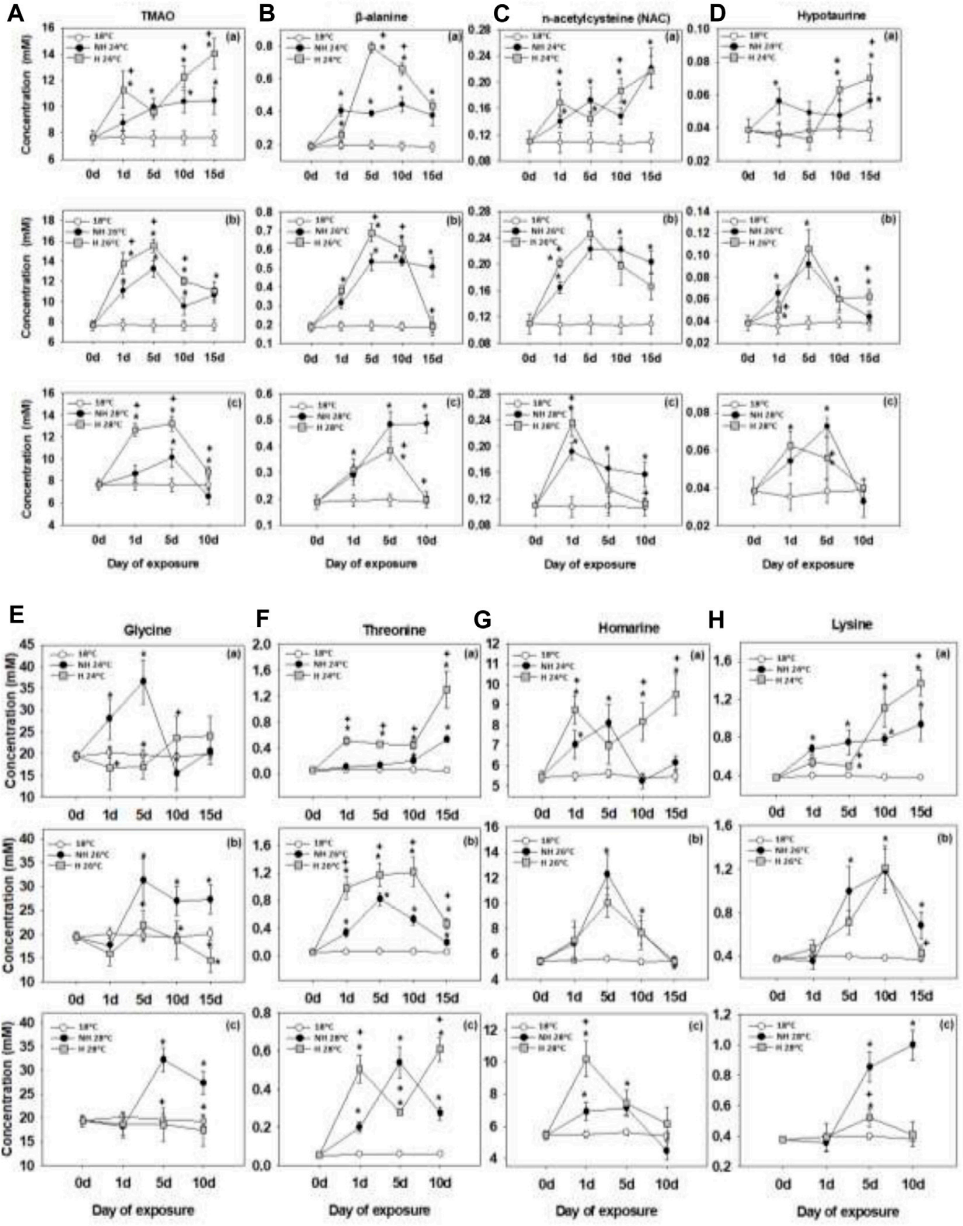
TMAO**(A)**, β-alanine **(B)**, n-acetylcysteine **(C)**, hypotaurine **(D)**, glycine **(E)**, threonine **(F)**, homarine **(G)** and lysine **(H)** levels in the mantle of heat-hardened (H) and non-heat-hardened (NH) *Mytilus galloprovincialis* mussels after exposure to 24°C **(a)**, 26°C **(b)** and 28°C **(c)** in comparison to acclimated at 18°C individuals (control). Values depict means ± SD of n = 8. Asterisk (*) depicts statistically significant changes (*p* < 0.05) when compared to control mussels, while cross (^+^) depicts statistically significant changes (*p* < 0.05) when compared to non-heat-hardened (NH) mussels.

At 24°C, levels oftrimethylamine-N-oxide (TMAO), β-alanine, n-acetylcysteine (NAC), hypotaurine increased throughout the exposure in the mantle of NH and H mussels. The concentrations of TMAO, β-alanine and hypotaurine were higher in the H mussels compared to the NH ones, particularly at the later stages (10–15 days) of exposure (Figures 5A–D). The levels of NAC did not consistently differ between the NH and H groups (Figure 5C).

At 26°C and 28°C the concentrations of TMAO, β-alanine, NAC and hypotaurine increased early on during warming and gradually declined after 5–10 days of exposure. Levels of TMAO were higher in the mantle of H mussels compared to the NH ones at 26°C and 28°C (Figures 5Cb, Cc). The concentrations of β-alanine and NAC tended were higher in the NH mussels compared to the H ones after prolonged (10–15 days) of exposure, with little difference between the groups observed at the earlier time points (Figures 5B,C). Mantle levels of hypotaurine were similar in the NH and H mussels throughout the exposure to 26°C and 28°C (Figures 5Db, 5Dc).

Glycine concentrations increased during warming in the NH mussels but remained near the baseline (control) levels in the H ones (Figure 5E). The warming-induced changes in threonine and homarine concentrations depended on the temperature with an increase throughout the exposure at 24°C and a transient peak after 1–5 days followed by a gradual decline at 26°C and 28°C (Figures 5F,G). A similar pattern was found for lysine concentrations except for the NH mussels at 28°C (Figure 5H). No consistent differences between the NH and H mussels in the concentrations of threonine, homarine and lysine were found.

### 3.6 Other metabolites

**FIGURE 6:**
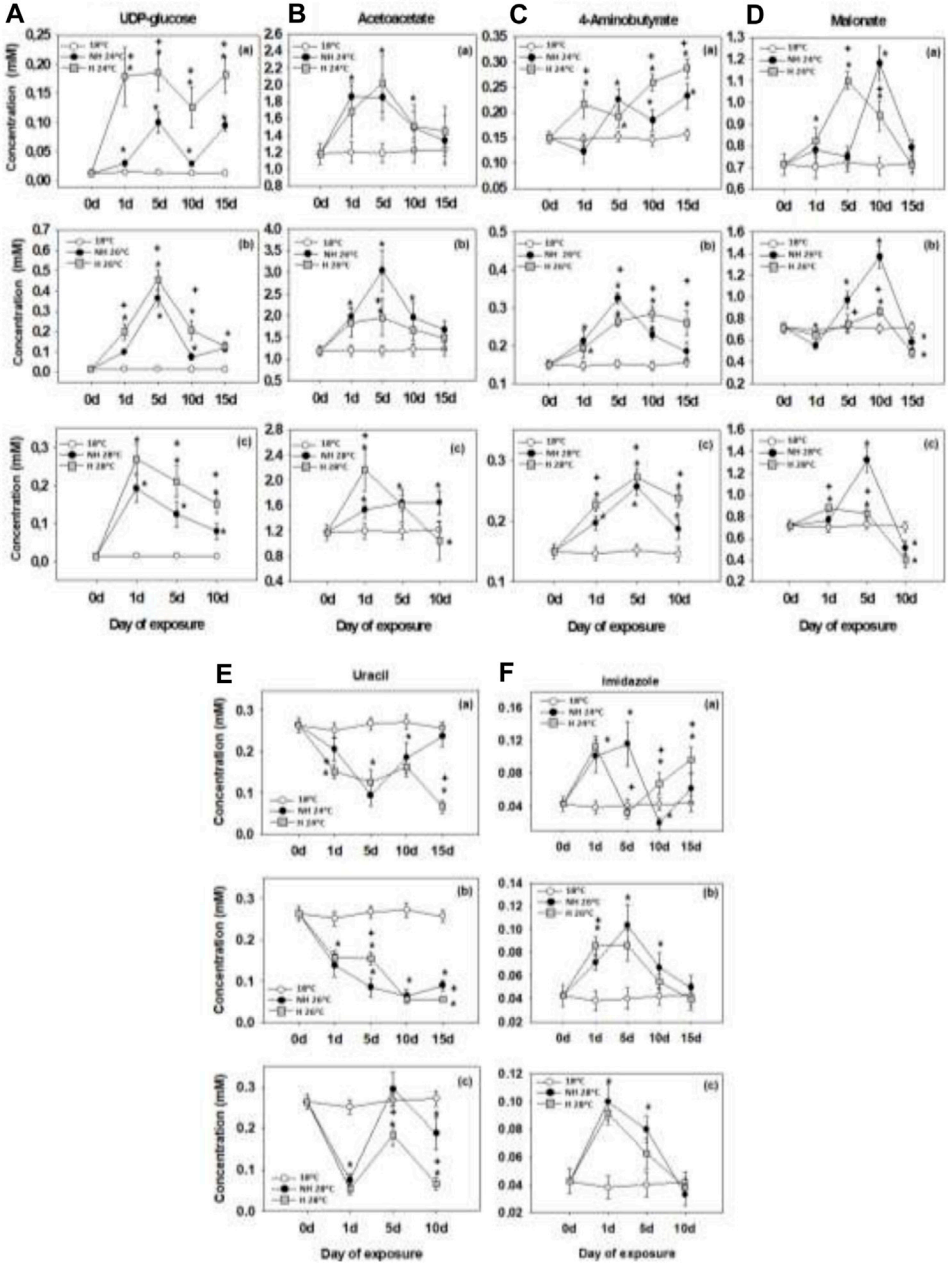
UDP-glucose**(A)**, acetoacetate **(B)**, 4-aminobutyrate **(C)**, malonate **(D)**, uracil **(E)** and imidazole **(F)** levels in the mantle of heat-hardened (H) and non-heat-hardened (NH) *Mytilus galloprovincialis* mussels after exposure to 24°C **(a)**, 26°C **(b)** and 28°C **(c)** in comparison to acclimated at 18°C individuals (control). Values depict means ± SD of n = 8. Asterisk (*) depicts statistically significant changes (*p* < 0.05) when compared to control mussels, while cross (^+^) depicts statistically significant changes (*p* < 0.05) when compared to non-heat-hardened (NH) mussels.

Concentrations of UDP-glucose in the mantle tissues increased during warming with higher levels observed in the H compared to the NH mussels (Figure 6A). The UDP-glucose levels peaked after 1–5 days of exposure and remained stable (24°C) (Figure 6Aa) or gradually decreased (26°C and 28°C) afterwards (Figures 6Ab, Ac).

Mantle levels of acetoacetate increased during the early (1–5 days) stages of warming and generally declined after 10–15 days, without any consistent differences between the NH and H groups (Figure 6B). Concentrations of 4-aminobutyrate increased during warming, with higher levels attained in the NH than in the H group after 10–15 days of exposure (Figure 6C). Tissue levels of malonate transiently peaked during warming, reaching higher peak levels in the NH than in the H mussels, and returned to the baseline (control) levels at 24°C or even lower values at 26°C and 28°C after 10–15 days of exposure (Figure 6D).

Mantle levels of uracil decreased during warming below the baseline (control) levels (Figure 6E). Concentrations of imidazole levels transiently increased after 1–5 days of exposure and decreased at the later time points reaching the baseline (control) levels after 10–15 days in most groups (Figure 6F).

### 3.7 Multivariate analyses of metabolite profiles

**FIGURE 7:**
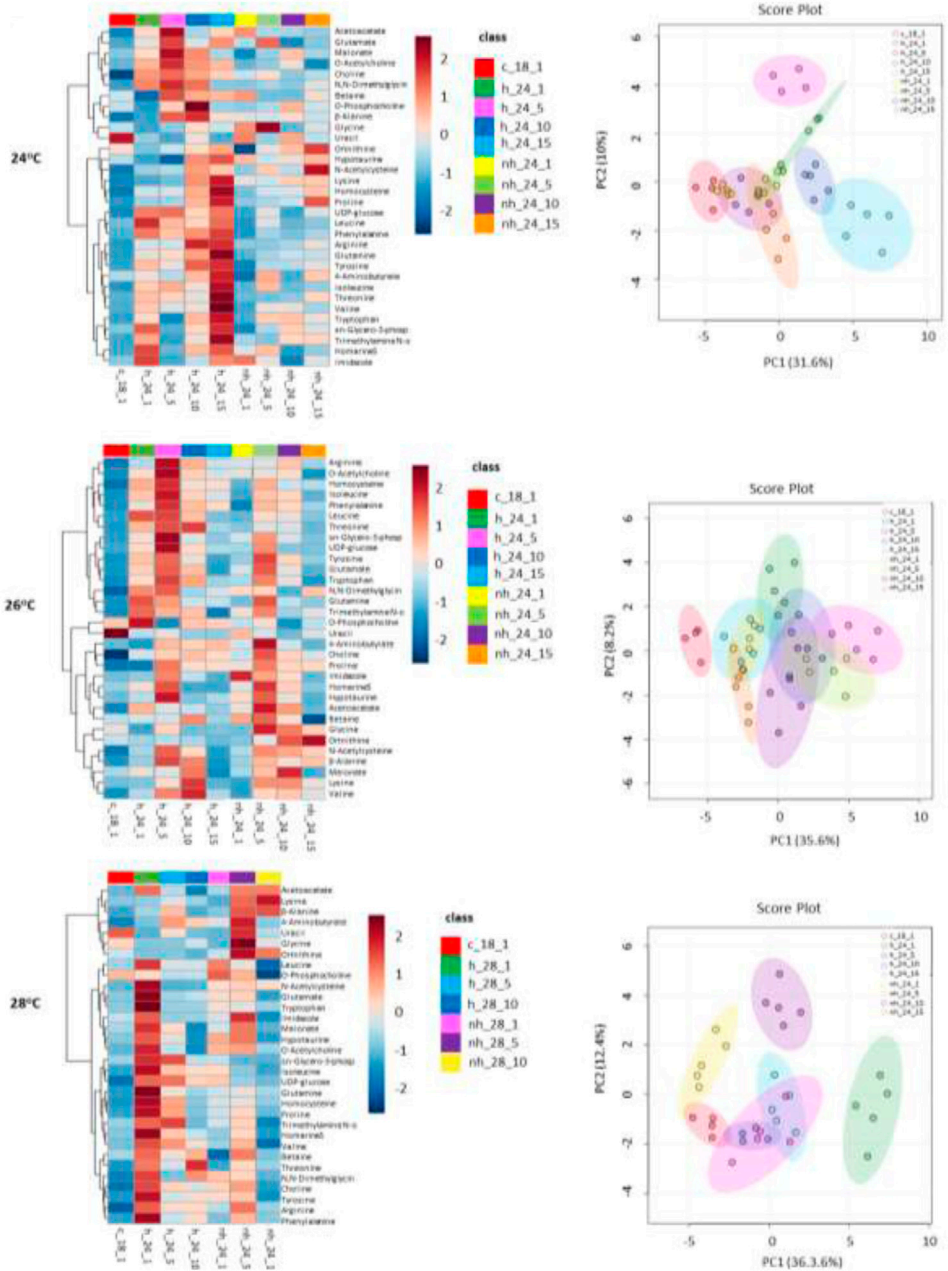
Heatmap depicting fold change of metabolites in the mantle of heat-hardened (H) and non-heat-hardened (NH) *Mytilus galloprovincialis* mussels after exposure to 24°C, 26°C and 28°C (dendrograms indicate the correlation between metabolites) and Correlations of metabolites with each of the first two principal components (PCs) in the multivariate analysis. (c_18_1: 18°C 1st day, h_24_1: hardening 24°C 1st day, h_24_5: hardening 24°C 5th day, h_24_10: hardening 24°C 10th day, h_24_15: hardening 24°C 15th day, nh_24_1: non-hardening 24°C 1st day, nh_24_5: non-hardening 24°C 5th day, nh_24_10: non-hardening 24°C 10th day, nh_24_15: non-hardening 24°C 15th day). (c_18_1: 18°C 1st day, h_26_1: hardening 26°C 1st day, h_26_5: hardening 26°C 5th day, h_26_10: hardening 26°C 10th day, h_26_15: hardening 26°C 15th day, nh_26_1: non-hardening 26°C 1st day, nh_26_5: non-hardening 26°C 5th day, nh_26_10: non-hardening 26°C 10th day, nh_26_15: non-hardening 26°C 15th day). (c_18_1: 18°C 1st day, h_28_1: hardening 28°C 1st day, h_28_5: hardening 28°C 5th day, h_28_10: hardening 28°C 10th day, nh_28_1: non-hardening 24°C 1st day, nh_28_5: non-hardening 28°C 5th day, nh_28_10: non-hardening 28°C 10th day).

The heatmaps in Figure 7 show the differences in the metabolome shifts between the non-heat-hardened (group NH) and heat-hardened (group H) mussels as well as between the different exposure temperatures. At 24°C, three groups of metabolites, showing correlated patterns of change, were identified based on the corresponding dendrograms. The first group included metabolites that increased towards mid-exposure in the H mussels (acetoacetate, glutamate, malonate, O-acetylcholine, choline, N,N-dimethylglycine, betaine, O-phosphocholine, β-alanine, glycine and uracil). The second group included metabolites peaking during the late exposure in both H and NH group (ornithine, hypotaurine, N-acetylcysteine, lysine, homocysteine and proline). The third group of metabolites increased during mid- and late exposure exclusively in the H group (UDP-glucose, leucine, phenylalanine, arginine, glutamine, tyrosine, 4-aminobutyrate, isoleucine, threonine, valine, tryptophan, sn-glycero-phosphate, trimethylamine, homarine and imidazole).

At 26°C, four groups of metabolites were identified. The first group showed a concentration increase during the first days of exposure in the H mussels (arginine, O-acetylcholine, homocysteine, isoleucine, leucine, phenylalanine, threonine), while those in the second group increased in both H and NH mussels (sn-glycero-phosphate, UDP-glucose, tyrosine, glutamate, tryptophan, N,N-dimethylglycine, glutamine and thimethylamine). Additionally, O-phosphocholine and uracil levels increased in the first days of exposure in the mantle tissue of the H group. The fourth group included metabolites peaking during the mid-exposure at both groups (4-aminobutyrate, choline, proline, imidazole, homarine, hypotaurine, acetoacetate, betaine, glycine, ornithine, n-acetylcysteine, b-alanine, malonate, lysine and valine).

At 28°C, the comparison between differently treated mussels revealed a group of metabolites (leucine, phosphocholine, N-acetylcysteine, glutamate, tryptophan, imidazole, malonate, hypotaurine, O-acetylcholine, sn-glycero-phosphate, isoleucine, UDP-glucose, glutamine, homocysteine, proline, trimethylamine, homarine, valine, betaine, threonine, N,N-dimethylglycine, choline, tyrosine, arginine, phenylalanine) that were elevated during the first day of exposure in the H group and during mid-exposure in the NH mussels, and a second group (acetoacetate, lysine, β-alanine, 4-aminobutyrate, uracile, glycine, ornithine) enriched at the mid- and late exposure in the NH group mussels.

(c_18_1: 18°C 1^st^day, h_24_1: hardening 24°C 1^st^ day, h_24_5: hardening 24°C 5^th^day, h_24_10: hardening 24°C 10th day, h_24_15: hardening 24°C 15th day, nh_24_1: non-hardening 24°C 1^st^ day, nh_24_5: non-hardening 24°C 5^th^ day, nh_24_10: non-hardening 24°C 10th day, nh_24_15: non-hardening 24°C 15th day).
(c_18_1: 18°C 1^st^day, h_26_1: hardening 26°C 1^st^ day, h_26_5: hardening 26°C 5^th^day, h_26_10: hardening 26°C 10th day, h_26_15: hardening 26°C 15th day, nh_26_1: non-hardening 26°C 1^st^ day, nh_26_5: non-hardening 26°C 5^th^day, nh_26_10: non-hardening 26°C 10th day, nh_26_15: non-hardening 26°C 15th day).
(c_18_1: 18°C 1^st^day, h_28_1: hardening 28°C 1^st^ day, h_28_5: hardening 28°C 5^th^day, h_28_10: hardening 28°C 10th day, nh_28_1: non-hardening 24°C 1^st^ day, nh_28_5: non-hardening 28°C 5^th^ day, nh_28_10: non-hardening 28°C 10th day).

PCA analysis was applied to statistically determine the differences in metabolite responses examined herein. According to the results for the different acclimation temperatures (24, 26, and 28°C), it seems that group H individuals were diverted in different clusters from both control and group NH individuals (Figure 7 right), mainly at 24 and 28°C, as well as at the first days (1-5) at 26°C. Specifically, at 24°C, PC1 explained 31.6% of the variance, while PC2 explained 10% (Figure 7A). At 26°C, PC1 explained 35.6% of the variance, while PC2 explained 8.2% (Figure 7B). Finally, at 28°C, PC1 explained 36.6% of the variance, while PC2 explained 12.4% (Figure 7C).

**FIGURE 8:**
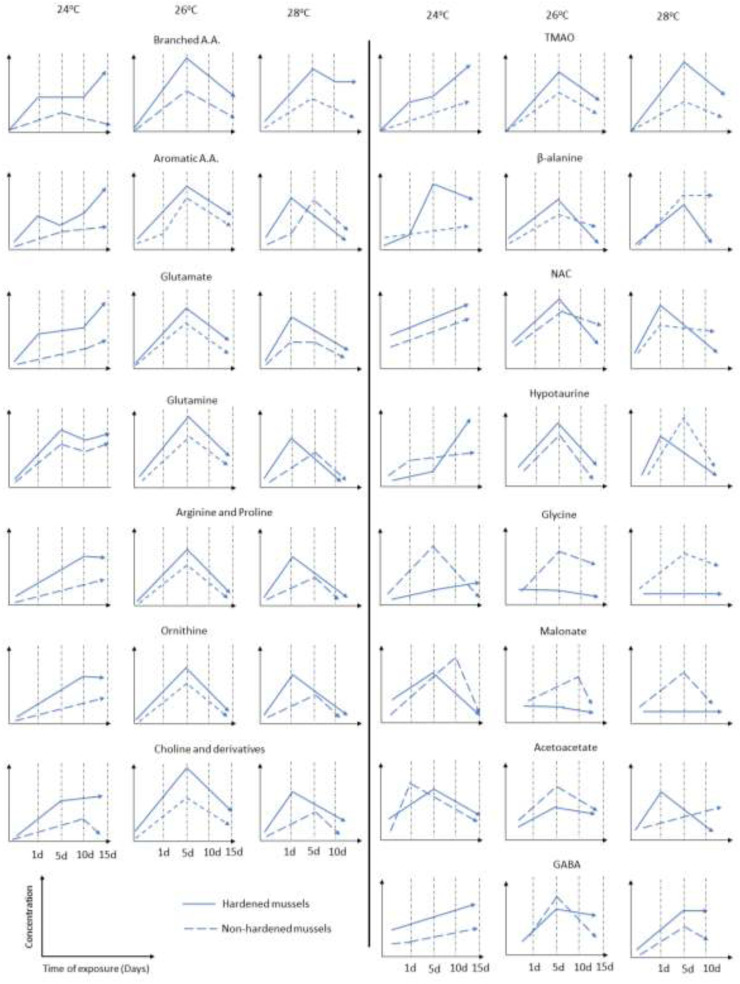
Trends and patterns of the accumulation of metabolites in non-heat-hardened (NH) and heat-hardened (H) *Mytilus galloprovincialis* mussels after exposure to different temperatures (24°C, 26°C and 28°C).

Finally, Figure 8 summarizes the trends of metabolite changes in the naïve and heat hardened mussels during exposure to different temperatures.

## 4 Discussion

### 4.1 Branched-chain amino acids

BCAAs, including leucine, isoleucine, and valine, are essential amino acids in mollusks and other animals (Fitzgerald and Szmant, 1997) and they play critical roles in cellular metabolism, growth, and stress signaling (Lynch and Adams, 2014). Moreover, BCAAs exert stimulatory effects on protein synthesis and inhibitory effects on proteolysis aiding protein deposition (Francesco and Enzo, 2017; Holeček, 2018). In addition to regulating protein turnover, leucine and isoleucine also play important roles in energy metabolism and redox homeostasis (D’Antona et al., 2010). Leucine increases the efficiency of mitochondrial ATP synthesis, which helps to mitigate oxidative stress (Wu et al., 2022). It also activates the mammalian target of rapamycin complex 1 (mTORC1), which stimulates biosynthesis, cell survival, and proliferation (Lynch and Adams, 2014). Isoleucine, on the other hand, protects against oxidative stress by regulating the peroxisome pathway and promoting acetyl-coenzyme A (acetyl-CoA) production (Wu et al., 2022). Thus the rapid and robust accumulation of BCAAs in H mussels during warming may have cytoprotective and enhanced thermotolerance observed in H mussels by improving ATP supply, protein synthesis (including heat shock proteins), and redox balance. On the other hand, BCAA accumulation was delayed in NH mussels and did not reach similar high levels as in the H mussels. Given that the mollusks cannot synthesize BCAAs (Fitzgerald and Szmant, 1997), the warming-induced accumulation of BCAAs in the mussels must reflect increased transport and retention of these amino acids from the dietary or waterborne sources (Wright and Pajor, 1989), or stimulation of BCAA production by associated microbiome (Lin et al., 2017).

The H mussels’ ability to maintain high BCAA levels was found to be dependent on the degree of thermal stress they were exposed to. When exposed to mild thermal stress (24°C), the H mussels were able to increase their BCAA levels throughout the 15 days of exposure. However, under more severe warming (26°C and 28°C), the capacity to retain high BCAA levels appeared compromised and the levels decreased to near-baseline levels after 10 or 15 days of exposure. The NH mussels showed a similar time course of BCAA changes at 26°C and 28°C (indicating that same regulatory mechanisms might be involved), albeit with lower absolute BCAA concentrations in the mantle tissues compared to the H group. The decline in the levels of BCAAs after prolonged and severe warming can make the cells of mussels more susceptible to oxidative stress and energetic imbalance (Flores et al., 1989; Nawabi et al., 1990). This vulnerability seems more pronounced in NH mussels that are less protected by BCAAs to begin with.

### 4.2 Aromatic amino acids

Aromatic amino acids (AAAs) like phenylalanine, tyrosine, and tryptophan are essential for mollusks, which cannot synthesize them and must acquire AAAs through their diet or from symbiotic organisms (Fitzgerald and Szmant, 1997; Parthasarathy et al., 2018). AAAs play a crucial role in stress tolerance as they serve as precursors for the biosynthesis of hormones and neurotransmitters (Han et al., 2019). Tyrosine and phenylalanine are involved in the biosynthesis of neurotransmitters and hormones such as dopamine, norepinephrine, and adrenaline, while tryptophan is involved in the biosynthesis of serotonin, melatonin, and kynurenic acid, which protects neurons from overstimulation (Lieberman et al., 2005; Zehra and Khan, 2014; Han et al., 2019). Heat hardening induced earlier and stronger accumulation of AAAs during warming in the mantle compared to findings in mantle tissue of the NH mussels. Like BCAAs, accumulation of AAAs cannot be due to the *de novo* synthesis and must reflect increased uptake from external sources or acquisition through protein recycling.

Generally, under mild thermal stress (24°C), H mussels showed earlier signs of AAAs accumulation and eventually reached higher levels of AAAs in the mantle tissues than the NH mussels. Under more severe thermal stress (26°C and 28°C), the main difference between the H and NH mussels was in the earlier onset of AAAs accumulation in the former group. After prolonged exposures at 26°C and 28°C, the AAAs concentrations converged on similar levels in both H and NH groups and declined towards the end (10–15 days) of exposures. At the highest studied temperature (28°C), the rate of this decline was faster in the H mussels compared with the NH counterparts. These trends indicate that the balance between the AAAs influx and their conversion to the secondary compounds like neurotransmitters and hormones is positive under the mild thermal stress allowing for a gradual increase of the pool of these essential amino acids, particularly in the H mussels. Given the important protective roles of AAAs and their derived secondary metabolites in regulation of redox balance, immunity and osmotic homeostasis (Lopez-Patino et al., 2013; Maitra and Hasan, 2016), robust accumulation of AAAs at 24°C might contribute to stress tolerance of H mussels. However, the capacity of the mussel tissues to balance supply and demand of AAAs appears to be exhausted after the prolonged exposures to severe thermal stress (26°C and 28°C). High rates of conversion of AAAs into the protective secondary metabolites (Georgoulis et al., 2021) apparently exceed the AAAs influx from diet and protein breakdown and indicates high cost of thermoprotection in mussels as the severity and duration of the thermal stress increases.

### 4.3 Glutamine family amino acid metabolism

The pathways involving amino acids of the glutamine family (arginine, glutamate, glutamine and proline) and their derivatives (like ornithine) play an important role in nitrogen metabolism, amino acid biosynthesis and production of signaling molecules like 4-aminobutyric acid (GABA) and nitric oxide (NO) (Newsholme et al., 2003; Zhang et al., 2017). Glutamine serves as a vital amino acid precursor and a non-toxic carrier of circulating ammonia (Yoo et al., 2020). In non-proliferating cells, glutamine is primarily used for protein synthesis or converted to glutamate, which serves as a precursor for α-ketoacids that fuel mitochondrial ATP production. Additionally, glutamate plays a role in synthesizing glutathione as well as non-essential amino acids like alanine, proline, aspartate, asparagine, and arginine (Yoo et al., 2020). Highly proliferative cells rely on glutamine for biosynthesis to generate precursors for amino acids, nucleotides, and fatty acids (Zhang et al., 2017; Yoo et al., 2020). The glutamine to glutamate ratio is a key indicator of cellular metabolic activity, as it decreases with increased glutamine consumption (as in highly proliferative cells), and increases with suppressed glutamine metabolism.

During warming, the concentration of certain amino acids from the glutamine family increased in the mantle of mussels of both groups. Glutamate, glutamine, arginine, and proline were all affected, but glutamate, compared to glutamine, saw a particularly strong accumulation relative to the baseline especially in H mussels. Strong glutamate accumulation in H mussels during warming might be due to the elevated pool of glutamine and BCAAs that can be converted in glutamate (Tamanna and Mahmood, 2014). High levels of glutamate accumulation indicates that warming-induced metabolic reprogramming is shifted toward enhanced ATP synthesis rather than proliferation (Zhang et al., 2017; Yoo et al., 2020). Moreover, high levels of glutamate support protection against oxidative stress by stimulating the synthesis of glutathione, particularly when combined with elevated levels of NAC, another GSH precursor, as found in our present study. This indicates activation of glutathione system and is consistent with an earlier report of upregulated glutathione reductase activity during thermal stress in *M. galloprovincialis*, with a particularly strong response shown by the H mussels (Georgoulis et al., 2021). Similar findings were reported in a sea cucumber *Apostichopus japonicus*, where elevated levels of glutamate were also observed during heat stress, along with the upregulation of proteins involved in glutathione metabolism (Xu et al., 2016).

The high glutamate pool in the warming-exposed mussels was associated with the accumulation of an important glutamate-derived metabolite, 4-aminobutyrate (GABA). GABA is a vital neurotransmitter in the central nervous system of invertebrates, including mollusks (Nguyen et al., 2021). Furthermore, the H group of mussels had significantly higher levels of GABA compared to the NH ones. This observation aligns with Dunphy et al.'s (2018) study, which also found higher levels of GABA in H than NH green-lipped mussels (*Perna canaliculus*). This increase in GABA levels suggests that the GABAergic synapse pathway is enhanced during the heat-hardening process, thereby retaining neural function and providing better endurance against thermal stress. Furthermore, the higher levels of glutamate in H mussels could potentially supply additional energy sources to the TCA cycle, thereby increasing GABA production through the GABA shunt (Bouche and Fromm, 2004; Singh and Roychoudhury, 2020).

The exposure of NH mussels at temperatures beyond 24°C resulted in accumulation of arginine and its downstream metabolites, ornithine and proline. The latter suggests that heat stress promotes activity of the arginine—ornithine—proline metabolic pathway, by increasing the activity of ornithine aminotransferase to generate D1-l-pyrroline-5-carboxylate (P5C) (O'Quinn et al., 2002). The increased levels of intracellular ornithine promote production of P5C, which can be used for proline biosynthesis (Ginguay et al., 2017). Irrespective of temperature exposure, in H mussels, no accumulation of ornithine was observed in the mantle tissue, despite accumulation of arginine and proline. Since the flux from ornithine to P5C is not favored under low intracellular concentrations of ornithine (Albaugh et al., 2017), it appears more likely that glutamate rather than ornithine serve as a source for proline synthesis in the H mussels. Proline accumulation can contribute to cytoprotection during warming stress since this amino acid can stabilize proteins and prevent their aggregation (Csonka, 1989; Liang et al., 2013). High levels of proline can also support the antioxidant defense of the H mussels during warming by scavenging ROS and increasing GSH pool (Zablocki et al., 1991; Ling et al., 2013). Accumulation of arginine in the H mussels, on the other hand, may be due to activation of the urea cycle for ammonium detoxification and as a source of fumarate it can fuel the TCA cycle (Georgoulis et al., 2022).

### 4.4 Choline and related metabolites

Choline plays a crucial role in maintaining cell volume, supporting nutrient metabolism, balancing osmotic conditions, and enhancing antioxidant capacity, thereby potentially mitigating the impacts of heat stress (Zablocki et al., 1991). In animals, choline can be obtained from phosphatidylcholine (PC) through phosphatidylethanolamine (PE) methylation (Reo et al., 2002). Exposure of mussels to heat stress resulted in increased choline levels. When faced with stressful conditions, the recycling of choline from PC breakdown is enhanced, allowing the organism to prevent complete choline deprivation (Li et al., 2005). Moreover, the elevated levels of related metabolites during heat stress, such as betaine, homocysteine, acetylcholine, and dimethylglycine, suggest that choline undergoes acetylation to form acetylcholine, a crucial neurotransmitter in animals (Wu and Hersh, 1994), and oxidation to betaine (Pritchard and Vance, 1981), instead of phosphorylation to PC, which leads to decreased PC concentrations. Betaine supports cellular stability and is vital for maintaining cellular function in stressed mussels (Sokolov and Sokolova, 2019). It also serves as a methyl group donor, directing methionine toward protein synthesis, and regulates gene expression through DNA or histones methylation (Zeisel, 2017; Alagawany et al., 2022). Additionally, betaine transfers a methyl group to homocysteine, resulting in the production of dimethylglycine and methionine, thereby sustaining the transmethylation cycle (Kidd et al., 1997).

The warming temperatures have led to an increase in homocysteine and dimethylglycine levels, indicating an upregulation of the transmethylation pathway that potentially contributes to increased methylation during heat stress. Within the transmethylation cycle, methionine produces S-adenosylmethionine (SAM), which acts as a methyl donor for the conversion of phosphatidylethanolamine (PE) to phosphatidylcholine (PC) catalyzed by phosphatidylethanolamine N-methyltransferase (PEMT) (Bremer and Greenberg, 1961; Li and Vance, 2008). This process generates three molecules of S-adenosylhomocysteine (AdoHcy) through the PEMT reaction, which are then hydrolyzed to adenosine and subsequently converted back to homocysteine (Kidd et al., 1997), while PC can be catabolized back to choline.

In the case of H mussels, exposure resulted in a faster accumulation of choline, betaine, acetylcholine, dimethylglycine, and homocysteine compared to NH. These metabolites reach higher levels at 24°C and on the first day of exposure above 26°C. The accelerated and stronger activation of these metabolic pathways appears to benefit the H individuals by enhancing neurotransmitter functions, improving cellular stability, maintaining osmotic balance, providing thermoprotection, boosting antioxidant capacity, and increasing methylation levels. Furthermore, during warming, H mussels significantly accumulated glycero-3-phosphocholine, whereas NH mussels showed no substantial increases in this metabolite except at lower levels at 26°C. Glycero-3-phosphocholine can be synthesized from PC and acts as an organic osmolyte, protecting cells against high concentrations of NaCl and urea (Gallazzini and Burg, 2009). This finding supports the hypothesis of an enhanced urea cycle in H mussels during heat stress. While the elevated levels of choline and its related metabolites were sustained through the entire 15 days exposure at 24°C in both H and NH mussels, exposure to higher temperatures (26°C and 28°C) curtailed this response after 5 days.

### 4.5 Organic osmolytes and cytoprotective compounds

Organic osmolytes, such as TMAO, NAC, hypotaurine, lysine, and β-alanine, play a crucial role in protecting the cellular structures of marine organisms, including mussels, from harsh environmental conditions such as warming (Mello and Barrick, 2003; Yancey, 2005; Rahman et al., 2016; Somero, 2022). In particular, TMAO and glycerylphosphorylcholine (GPC) act as effective protectants, enhancing protein stabilization and reducing protein denaturation caused by heat or chaotropic compounds like urea (Mello and Barrick, 2003; Yancey, 2005; Rahman et al., 2016; Somero, 2022). Likewise, lysine contributes to protein stabilization by binding to denatured proteins (Lin and Timasheff, 1996), while β-alanine supports redox balance, further aiding in protein stabilization (Bishop et al., 1983). The accumulation of hypotaurine and NAC enhances cellular cytoprotection in mussels by acting as antioxidants. Hypotaurine scavenges harmful radicals, such as HOCl and OH radicals, thereby yielding taurine (Aruoma et al., 1988) that accumulates during heat stress in *M. galloprovincialis* (Georgoulis et al., 2022). NAC improves the activity of oxidative stress enzymes, bolstering oxidative defense mechanisms under elevated temperatures (Maksimchik et al., 2008) and helps prevent DNA damage and resists apoptosis (Zhao et al., 2021). NAC also serves as a precursor of glutathione, enhancing detoxification mechanisms (Peña-Llopis et al., 2014). Therefore, a faster and more pronounced accumulation of these protective compounds in the H mussels could enhance their ability to stabilize the proteome and reduce protein denaturation caused by heat stress and urea accumulation. Furthermore, preferential accumulation of these protein-stabilizing osmolytes at the expense of the less compatible amino acids like glycine may enhance this effect (Somero, 2022).

The continuous accumulation of organic osmolytes at 24°C until the 15th day of exposure contributed to the cellular cytoprotection of H mussels. However, prolonged exposure to higher temperatures, 26°C and 28°C, led to depletion of the osmolyte pool after the 5th and 1st day, respectively, resulting in a decrease in the mussels’ cytoprotective capacity. At these severe thermal stress levels, the stabilizing effects on macromolecules and antioxidant mechanisms were superseded by stronger mechanisms like heat shock proteins (HSPs) and antioxidant enzymes, which remaind elevated throughout the exposure to severe thermal stress (Georgoulis et al., 2021).

### 4.6 Other metabolites

In both H and NH mussels, temperature increase has led to increased levels of acetoacetate, a ketone body synthesized from acetyl-CoA as the end product of fatty acid metabolism (Laffel, 1999). Heat-stressed mussels may stimulate lipid metabolism, which can serve as an energy source when glucose availability is limited (Laffel, 1999; Tian et al., 2015; Fan et al., 2019). In the present study, acetoacetate levels increased without significant differences between H and NH mussels at 24°C and 26°C. At 28°C, however, H mussels exhibited a faster increase in ketone metabolism compared to NH individuals suggesting a stronger stimulation of lipid metabolism in mantle.

Heat stress also caused the accumulation of malonate in mussels. Malonate acts as a competitive inhibitor of the enzyme succinate dehydrogenase, a key molecule in the tricarboxylic acid (TCA) cycle and electron transport chain (Kim, 2002). Increased malonate levels can negatively impact energy production by reducing the activity of the TCA cycle and limiting cellular respiration capacity (Zampiga et al., 2021). The accumulation of malonate, particularly in NH mussels at all elevated temperatures, indicates a restriction of energy production during heat stress, contrasting with H mussels that exhibited a significant increase in malonate only on the 5th day at 24°C. This observation is closely related to the metabolic depression observed in *M. galloprovincialis* when acclimated to 24°C, accompanied by extended periods of valve closure (Anestis et al., 2007). Additionally, the increased concentrations of succinate and fumarate in NH mussels support the hypothesis of anaerobic succinate metabolism, as malonate inhibits the activity of succinate dehydrogenase. In contrast, the lower concentrations of malonate in H mussels at 26°C and 28°C, compared to NH ones, align with previous findings that demonstrated no elevation of succinate and fumarate, indicating a more robust aerobic pathway in the TCA cycle and a delayed switch to anaerobiosis (Georgoulis et al., 2021).

UDP-glucose (UDP-Glc) exhibited increased concentrations at temperatures beyond 24°C. UDP-Glc is produced through glycogenesis and participates in the biosynthesis of glycogen, glycoproteins, glycosaminoglycans, and glycosphingolipids (Durrant et al., 2020). Under stressful conditions, bivalves prioritize carbohydrates as their primary energy source, with fatty acids serving as a secondary source (Erk et al., 2011). The accumulation of UDP-Glc in thermally stressed mussels indicates higher demands for glycogen production during warming, which has been attributed as a metabolic adaptive strategy against heat stress (Maazouzi et al., 2011). Specifically, this phenomenon was particularly pronounced in H mussels compared to NH ones, with significantly higher UDP-glucose levels at all elevated temperatures, indicating enhanced glycogenesis rates and increased production of energy sources like glycogen to withstand acute thermal stress.

## 5 Conclusion

Our study provides insights in the metabolic mechanisms that may lead to increased tolerance of *M. galloprovincialis* against increased seawater temperatures. Specifically, metabolic rearrangement induced by heat hardening appears to enhance the thermal protection *via* increase of particular metabolites. Most notably, these metabolites included BCAAs, AAAs, and compounds with protein stabilizing and antioxidant properties. However, metabolites exhibited different patterns of accumulation at different temperatures indicating the interplay of several metabolic pathways during heat stress. As temperature rose, the distinction between the responses of H and NH mussels decreased progressively. This was demonstrated by hierarchical clustering analysis, which revealed that at 24°C, 26 metabolites were preferentially enriched in the H mussels, while at 26°C, this number reduced to 9 only. At 28°C, a sizable group of 24 metabolites increased in both treatment groups, albeit with an earlier onset in the H mussels. These findings suggest that with increasing thermal stress, similar protective metabolic mechanisms are activated in both groups. However, the H mussels exhibited an earlier and stronger activation of this conserved protective response, contributing to enhanced thermal protection. The latter is clearly depicted by the fact that the NH mussels exhibit a 100% mortality at 28°C by the 10th day of exposure, in comparison to the H individuals. Notably, the majority of metabolites exhibited a biphasic response peaking within 5 days of warming in H and NH mussels. We propose that the initial increase in metabolite levels during the first 5 days serves as a preemptive defense mechanism to safeguard the structure and functionality of proteins, prior to the sufficient expression of molecular chaperones like HSPs that provide protection during the later phase. This highlights the potential of heat hardening as a highly promising approach in combating the impacts of global warming on cultivated and economically valuable bivalves, providing increased endurance to thermal stress. However, the energy costs associated with this response and its potential benefit on mussels’ thermotolerance during prolonged exposure to elevated temperatures remain uncertain.

## Data Availability

The raw data supporting the conclusion of this article will be made available by the authors, without undue reservation.
